# Nurse managers’ leadership styles as an impetus to patient safety in an academic hospital

**DOI:** 10.4102/hsag.v28i0.2344

**Published:** 2023-10-25

**Authors:** Virgina S. Palweni, Jacobeth M. Malesela, Moreoagae B. Randa

**Affiliations:** 1 Department of Nursing Science, School of Health Care Sciences, Sefako Makgatho Health Sciences University, Pretoria, South Africa; 2 Department of Public Health, School of Health Care Sciences, Sefako Makgatho Health Sciences University, Pretoria, South Africa

**Keywords:** academic hospital, leadership styles, nurse manager, patient safety, perceptions

## Abstract

**Background:**

Successful implementation of patient safety plans in a hospital necessitates, among other things, the leadership capacity of nurse managers. Patient care delivery errors and adverse events continue to occur for a variety of reasons, including a failure to follow recommended patient safety strategies. Certain leadership styles foster interactions with healthcare staff, resulting in work environments that promote positive patient outcomes. It is unclear what nurse managers believe about the type of leadership style that drives patient safety.

**Aim:**

The goal was to explore the nurse managers’ perceptions of leadership styles as an impetus to patient safety.

**Setting:**

The study was conducted at an academic hospital in the Tshwane District of Gauteng province.

**Methods:**

A qualitative exploratory and descriptive contextual design was used. Semi-structured face-to-face interviews were conducted with 20 purposefully selected nurse managers. A thematic data analysis method was used to analyse data.

**Results:**

Common leadership styles among nurse managers and challenges affecting the efficiency of nurse managers’ leadership styles emerged as themes.

**Conclusion:**

Nurse managers must have appropriate leadership styles to improve patient safety. Human and material resource shortages, as well as a lack of collaborative practice among healthcare professionals, jeopardise nurse managers’ ability to maximise patient safety.

**Contribution:**

The article provides insight into nurse managers’ perceptions of leadership styles as critical to improving patient safety. Recommendations included the need for a structured ongoing leadership training programme to develop and strengthen the skills of newly appointed and existing nurse managers.

## Introduction

The World Health Organization (WHO 2021) regards patient safety framework as interdisciplinary in nature. Patient safety is described as a framework of organised activities that creates cultures, processes, procedures, behaviours, technologies and environments in healthcare that consistently and sustainably lower risks, reduce the occurrence of avoidable harm, make errors less likely and reduce the impact of harm when it does occur. Patient safety is also a strategic priority for modern healthcare and is central to countries’ efforts in working towards Universal Health Coverage (WHO 2020). Furthermore, it will not be worth achieving if healthcare itself poses a threat to people’s health.

Again, the WHO (2021) claims that 1 in 10 patients is subjected to an adverse event while receiving hospital care in high-income countries, whereas about 134 million adverse events because of unsafe care occur in hospitals in low- and middle-income countries, contributing to around 2.6 million deaths every year. Also, regarding the reported patient safety occurrences in a long-term rehabilitative hospital in KwaZulu-Natal posit that about 4.12 incidents per 10 000 inpatient days were reported whereby 52% of the adverse health events occurred in females aged between 50 years to 59 years, Mgobozi and Mahomed (2021). The latter authors furthermore state that about 96% of incidents were reported during the day and 33% within the shift change. The commonly reported patient safety incidents are pressure ulcers, falls, injuries, hospital-acquired infections, and medication errors (Mgobozi & Mahomed, 2021).

The international and national patient safety initiatives are in place to eliminate and/or monitor avoidable harm because of unsafe healthcare such as the global patient safety action plan (WHO 2021), national guidelines on patient safety incident reporting and learning in the Health Sector of South Africa (National Department of Health 2022). Leadership style is the crucial component of the successful implementation of a patient safety programme that cannot be delegated (Mayeng & Wolvaardt 2015). Utari et al. (2020) claim that it is not possible for the holder of the highest leadership position to manage, supervise and coach all employees in the hospital. Leadership styles are shared across the macro- and micro-levels according to the specific services such as medical and nursing.

Consequently, public hospitals in some of the countries such as Indonesia categorised the nurse managers into five hierarchical levels for effective nursing service management. Such categories include the first-line nurse managers who are considered lower-level managers, area managers and heads of sub-nursing departments being considered middle managers, and the heads of nursing departments and the directors or vice directors of nursing considered top managers (Gunawan et al. 2020).

The nurse manager’s leadership style, in particular, plays a vital role in affecting outcomes for healthcare organisations, personnel, and patients (Wang & Dewing 2021). Effective leaders possess the necessary individual skills such as communication and creation of a healthy work environment, and collaboration and the traits that include trustworthiness, commitment, and open-mindedness (Cummings et al. 2021).

Effective leadership styles influence nurses’ well-being, staff retention and care delivered to patients. Contrastingly, ineffective leadership style contributes to poor well-being, stress or burnout among nurses, which impacts the provided care and consequently affects patient safety outcomes, such as medication errors or patient incident (Cummings et al. 2021). During clinical accompaniment, the researchers observed how nurse managers responded to issues of patient safety in several wards. For example, basic hand washing in between patients during the provision of care was not practiced by some of the healthcare practitioners. According to Mehta et al. (2014), hospital-acquired infections are a major cause of increased mortality and morbidity rates in hospitals. Adding to the problem is the evidence of inaccurate documentation of care such as intake and output and medication administered (Mehta et al. 2014). A lack of insight into patient safety and an inability to work in a collaborative team among some of the nurse managers were noticed. It is in the light of these challenges that the study sought to explore the perceptions of the nurse managers’ leadership styles as an impetus to patient safety.

## Objective and the research question

The objective was to explore and describe nurse managers’ perception of leadership styles as an impetus to patient safety. One open-ended question was asked, namely what are your perceptions regarding the nurse managers’ leadership style as an impetus to patient safety?

### Research method and design

The study used a qualitative exploratory and descriptive research design that is contextual in nature. The approach and design of choice was deemed to be appropriate for the study because nurse managers as people working together are actively engaged in creating and restructuring meanings through their daily interactions as leaders to facilitate patient safety in an academic hospital. The qualitative, exploratory and descriptive research design was used to delve in depth their perceptions through face-to-face, semi-structured interviews. The approach used demands bracketing whereby the researcher also declares personal biases and assumptions and puts them aside to increase objectivity of the study.

### Researcher characteristics and reflexivity

The researcher is a preceptor appointed by the university to facilitate work-integrated learning of medical students placed at the academic hospital during clinical blocks. The researcher assumed that the views, insights, and meanings assigned to their leadership style that enhances patient safety culture differ and are unique to individual nurse managers. Furthermore, the participants willingly and honestly shared their perceptions with the researcher in depth.

### Research setting and context

The study was conducted at a selected academic hospital in Tshwane. The hospital is divided into 44 wards and 10 outpatient departments (OPDs), with 1652 approved beds and 1370 usable beds. The hospital under study is a level three hospital and serves a 1.7 million population with catchment areas that include Bojanala District in the North-West province and Limpopo province. The hospital could not cater for the population that has increased significantly. There is an abnormal referral route because of a lack of tertiary and regional institutions in the cluster, which led to the nursing staff suffocating as a result of high volumes of workload that resulted from overcrowding. Overcrowding of patients and limited infrastructure led to unavailability of beds, which in turn resulted in patients not being properly monitored according to the set standards, as well as difficulty to sustain infection control protocols. Consequently, such circumstances compromised the managers’ leadership styles to drive patient safety.

### Study population and sampling strategy

The population is the nurse managers, composed of a director nursing services (DNS), deputy directors (DD), assistant directors (ASD), generalist nursing service managers (NSM), operational managers, generalist and operational managers – specialists (OPM-specialist or OPM-generalist). According to the hospital records, there are a total of 68 nurse managers as indicated in Table 1.

**TABLE 1:** Categories of nurse managers.

Category	Number
Director: Nursing services	1
Deputy directors	19
Assistant directors	4
Nursing service managers	7
Operational manager – generalist	5
Operational manager – specialist	33

**Total**	**68**

Recruitment was performed by the researchers during the manager’s monthly meetings, as they assembled in one room. Participants were informed about the study, its purpose and its significance. The ethical principles were also discussed. Participants were furthermore reassured that their names will not be used, but instead code numbers were assigned to comply with the *Protection of Personal Information Act* (POPIA). Purposive sampling was used to select 20 participants who are duly registered with the South African Nursing Council and have been appointed as nurse managers for 3 years and more. The nurse managers signed the informed consent to indicate their willingness to participate and share their perceptions, as well as for the recorded interviews. Based on their exposure period, the targeted nurse managers were able to provide rich relevant information about the phenomenon of concern (Fouché, Strydom & Roestenburg 2021). The sample consisted of a director, five deputy directors, one assistant directors, five nursing service managers, three generalists and five specialist’s operational managers.

### Data collection method

The researcher began by negotiating an entry, followed by interviews and the establishment of rapport with the participants. The procedure allowed the researcher to maintain contact with the appropriate authorities (Fouché et al. 2021). The researcher kept the relationship going by sticking to the agreed-upon data collection schedules.

The first part of the semi-structured interviews included enquiry about the participants’ sociodemographic information, and the second part included one open-ended question (What are your perceptions of leadership styles as an impetus to patient safety?) with a series of probing questions such as ‘When you say …, what do you mean?’ to elicit clarity from their initial response in order to obtain in-depth data about the studied phenomenon. An interview guide was used to collect data; in addition, field notes were taken during the process of the interviews. Furthermore, with permission from the participants, an audio recorder was used. Face-to-face, semi-structured interviews were conducted in English using a preprepared and pretested interview guide. The participant prearranged the time and location, which was agreed upon with the researcher. Measures were put in place at the venue to eliminate disruptions during the interview process. All interviews were conducted in a safe, quiet room with a ‘do not disturb’ sign outside the door.

Before each interview, participants were given numbers to replace their names, and they were reminded to maintain their anonymity, confidentiality and rights throughout the research. Participants were encouraged to speak up and share their personal experiences in their own words. The primary researcher conducted each interview, which lasted 45 min to an hour. At the end of each interview, the researcher reminded the participants of the need for a second contact with them via phone calls to schedule another meeting to verify if the findings were a true reflection of what the participants said while answering the study’s research question.

Data collection continued until data saturation was reached, as evidenced by information repetition at the 15th interview session. Two additional interviews were conducted to confirm that no new data had emerged, yielding an exact sample size of 17 participants. Data were collected between 21 October 2019 and 19 November 2019.

The researcher made certain that each recorded interview was listened to multiple times to get a sense of the overall picture and then transcribed verbatim within 24 h while the researcher had fresh recollection of all the events that occurred during each session. The transcript was updated to include the field notes. The transcribed data and audio notes were saved in a password-protected electronic file that only the researcher and the supervisor had access to.

### Data analysis method

Tesch’s 2019 thematic approach was used for data analysis (Creswell 2014). The researcher went through the transcripts one by one, reading each one carefully and repeatedly to get a sense of the information. The transcripts were examined one by one to find the underlying meaning in the texts. As ideas came to mind, notes were made in the margins of the documents. The development of a method for classifying and indexing data is a critical step in data analysis. The data were organised into manageable categories.

The researcher then compiled a list of all the themes and subthemes and grouped them together. The data’s themes and subthemes were abbreviated as codes, which were written next to the appropriate text segments. The codes allowed the researcher to see if any new codes or categories emerged. Additionally, descriptive wording was attached to the so-called meaning areas and topics, and the data were classified. The researcher created the initial analysis by combining all the data from each category. The transcripts were analysed by an independent coder who is an expert in qualitative data analysis. To discuss and consolidate the themes, a consensus meeting was held to increase the rigour of the data (Creswell & Creswell, 2018).

### Measures of trustworthiness

The researcher conducted 45 min – 60 min interviews with the participants, allowing for extended engagement with the participants. The researcher maintained an audit trail of all documentations pertaining to the research study, such as all audiotaped material, written notes and verbatim transcriptions. The field notes were used to clarify the nurse manager’s perceptions, and they were included in the study’s audit trail. Participants who met the inclusion criteria were interviewed until the data were saturated.

Direct quotations from nurse managers were used to supplement the description of their leadership perceptions that drive patient safety. To strengthen the validity of the research findings, an expertly chosen independent coder was appointed to analyse the data. The researcher and the independent coder met to reach an agreement on preliminary end results. The researcher presented the findings of the study to all participants, who confirmed that they were descriptive of their perceptions.

### Ethical considerations

Ethical clearance to conduct this study was obtained from the Sefako Makgatho Health Sciences University Research and Ethics Committee (No. SMUREC/H/281/2019:PG). Permission to conduct the study was obtained from the chief executive officer and the appropriate director for nursing services at the academic hospital where the study was conducted. Confidentiality and anonymity, and the right to protection of participants from discomfort and harm were ensured throughout the study. The actual names of the participants and that of the hospital were replaced by pseudonyms. To minimise personal bias and assumptions, the researchers provided an audit trail, which highlighted every step of data analysis to provide a rationale for the decisions made. The recorded interviews and transcripts were kept under lock and key and are only accessible to the researcher and the supervisors involved in the study, and will be destroyed after 5 years following the publication of the study to uphold confidentiality and anonymity.

With reference to the informed consent, the researcher was responsible to convey information about the study and its purpose and objectives truthfully to the participants in a way that they could comprehend to facilitate the authenticity and quality of the research outcomes (Fouché et al. 2021). Hence, the study purpose and objectives of the study were explained to the participants. The researcher allowed the participants time for a question-and-answer session to clarify issues or concerns and waited 5 min – 10 min after the information session before requesting the participants to sign the consent form indicating their willingness to participate in the study voluntarily.

With the right to self-determination, participants were informed of their right to discontinue their participation at any time during the process without any prejudice. The participants were not exposed to any physical or financial harm during the data collection process (Brink et al. 2018; Gray, Grove & Sutherland 2022).

## Results

Seventeen (*n* = 17) female nurse managers with the age ranging from 35 to 60 years were interviewed at the data saturation point. The mean age was 55 years. The categories consisted of one director of nursing services, three deputy directors, six assistant directors and seven operational managers of which two of them did not have an additional qualification in health service management and clinical specialisation. The participants provided rich insights into their leadership styles they commonly engaged in, including the challenges frustrating the efficiency of their leadership in patient safety. Consequently, proposed measures can be implemented to maximise their leadership styles. A total of two themes each with three subthemes emerged and are summarised in Table 2.

**TABLE 2:** Themes and subthemes on the nurse managers’ leadership styles as an impetus to patient safety.

Themes	Subthemes
1. The common leadership styles among nurse managers	1.1. Autocratic leadership 1.2. Laissez-faire leadership 1.3. Transformational leadership
2. The challenges affecting efficiency of nurse leadership	2.1. Shortages of nurses 2.2. Shortages of supplies and equipment 2.3. A lack of interprofessional collaboration

### Theme 1: The common leadership style among nurse managers

The nurse managers have insight into different leadership styles that they may exhibit from one situation to another as an effort to drive patient safety. Such styles include autocratic leadership, laissez-faire leadership and transformational leadership which emerged as subthemes.

#### Subtheme 1.1: Autocratic leadership

One of the nurse managers was uneasy about other colleagues’ persistent use of an autocratic leadership style and its related effects on employees and echoed the notion that:

‘Some of us when they have work pressure, tend to stick to top-to-bottom approach commanding and controlling the staff members creating a situation where their word is final.’ (Participant 8, 58 years, ASD)

‘A colleague hides behind being strict and set up clear directions and advice which they expected to get from others, for example, their own subordinates especially if does not have the latest knowledge of leadership, they demand that there should be clear rules and laws for everything and there is no flexibility.’ (Participant 13, 42 years, NSM)

#### Subtheme 1.2: Laissez-faire leadership

Seemingly, the nurse managers in sharing their perceptions suggested that some of their colleagues tend to be indecisive, would leave decisions to subordinates and provide limited direction, as evidenced by the citation from the three participants:

‘Sometimes it feels like everybody is in charge in some areas in the hospital. When issues do occur, they are often solved reactively of which in a clinical situation can be less than ideal.’ (Participant 2, 42 years, NSM)

‘Such colleagues constantly remain aloof and lead remotely. It is business as usual as their responsibility is to ensure that their managerial tasks are delegated to the lower management and is implemented without their leadership or follow-up feedback.’ (Participant 6, 58 years, NSM)

‘Ijooh! The colleague is normally hands-off and allow group members to make the decisions, and let them set their own deadlines as a result, late or no submission of important reports and supplies requisitions.’ (Participant 15, 48 years, NSM)

#### Subtheme 1.3: Transformational leadership

Additionally, in the process, nurse managers acknowledge that for them to drive patient safety, they should take cognisance that employees, in particular the nurses, have unique abilities and expectations that should be met as evidenced by:

‘I must admit one can always try to reach out and support employees in the endeavor to advance their careers; however, one should be able to align their development to the hospital needs as a succession plan.’ (Participant 8, 58 years, ASD)

‘Patient overflow in the wards and constrained resources create a vicious cycle of preventable patient-safety-related incidences and that is scary. This calls for our creative thinking and change the way we manage and lead patient care to maximise safety.’ (Participant 10, 60 years, DD)

‘In my area we have these small things to pep up staff, for example, such as birthdays surprise gifts as a recognition in order to make them feel they belong.’ (Participant 4, 58 years, ASD)

### Theme 2: The challenges affecting efficiency of nurse leadership styles

The nurse managers perceived challenges that affected the efficiency of their leadership to drive patient safety. The perception is that leadership is a shared encounter among multidisciplinary team with adequate resources. Perceived challenges are shortages of nurses, shortages of supplies and equipment and a lack of interprofessional collaboration.

#### Subtheme 2.1: Shortage of nurses

When participants were alluded to the progressive depletion of nurses, they said:

‘We have vacancies of retired, resignations of staff for whatever reason, who are not replaced and no new appointments. While the situation embarrasses our role as managers to promote quality care, the expectation is that the remaining cadre of nurses stretch themselves beyond the limits.’ (Participant 8, 58 years, ASD)

‘Ijooh! The red tape around the process related to advertising of nursing vacancies and appointments is frustrating because nurse managers’ inputs fall on deaf ears.’ (Participant 4, 58 years, ASD)

‘Set up the nurses to absent themselves from work, burn out leading to poor nursing-care-related issues like lack of recording, increasing medical-related errors and strained relations.’ (Participant 5, 50 years, NSM)

#### Subtheme 2.2: Shortages of supplies and equipment

Inadequate shortages of supplies and equipment interfere with the implementation of patient safety plans. This is what the participants said:

‘What is frustrating, for example, is that the hospital bought new linen and patient gowns and towels. Because of the centralised laundry service, we end up with inadequate supplies of bed linen, patient gowns of some are torn and cannot be used on patients or covering beds and pillowcases.’ (Participant 8, 58 years, ASD)

‘Currently our challenge is not only about staffing, we have shortages of all types of resources. Instead, we receive equipment that nobody knows of.’ (Participant 17, 60 years, DNS)

#### Subtheme 2.3: A lack of interprofessional collaboration

Nurse managers perceived that working in silos affected patient safety. They needed responsibilities with other health professionals involved for problem-solving, and make the decisions needed to formulate and carry out plans for safe patient care:

‘Professional teams are not integrated, and we do no work as partners that seems to be working towards ensuring initiatives enhance the safety of patients.’ (Participant 4, 58 years, ASD)

‘Inability to work harmoniously as a team, lack of cooperation and the staff attitudes.’ (Participant 9, 55 years, OPM Specialist)

‘There are no combined multidisciplinary team platforms for discussion of reported patient safety related incidences to enable team members to contribute towards preventive measures.’ (Participant 12, 52 years, NSM)

## Discussion

The leadership style of nurse managers is influenced by a variety of factors, including the organisation, the situation and the employees. Nurse managers’ leadership styles have an impact on the organisational climate, patient safety and job satisfaction. According to Fries, Kammerlander and Leitterstorf (2021), leadership styles and behaviours are defined as ways in which the leader influences the behaviour of the employees to provide directions and goals through motivation and the definition of rules. Recognising different leadership styles allows managers to develop skills to become better leaders while also improving relationships with colleagues who were previously difficult to work with (Xu 2017). Autocratic leadership, laissez-faire leadership and transformational leadership are all styles used by nurse managers to drive patient safety.

Autocratic leadership styles may be useful in situations where decisions must be made quickly and decisively. When the autocratic style is dominant, the leader will check the employees and give them negative feedback, which may be accompanied by open anger and punishment. Employee performance suffers as a result of this (Kalu & Okpokwasili 2018).

Laissez-faire leaders, in contrast to the previously discussed style, are hands-off, avoid making decisions, ignore responsibilities, and do not exercise authority (Sfantou et al. 2017). Dominant use of such a leadership style contributes to increased job stress and anxiety, affecting work performance and retention, as well as increasing medical errors and adverse patient care events (Khan & Tidman 2021). According to Daly et al. (2014), in a complex hospital like the one under study, clinician engagement must be supported by a leader who is resourceful and involved and takes responsibility to drive patient safety.

Participants believe that transformational leadership not only improves patient safety outcomes but also nurtures and motivates staff members. Similarly, Wong, Cummings and Ducharme (2013) contend that transformational leadership influences positive patient safety outcomes such as fewer adverse events and complications. Furthermore, as transformational leaders, managers recognise that employees have unique abilities and expectations, and they spend time teaching and coaching employees to help them develop. Additionally, within the constraints of resources, managers create a positive practice environment (Boamah et al. 2018).

Furthermore, according to Kvist et al. (2013), failure of leadership to create a conducive work environment eventually harms patients. Transformational leadership appears to be the best style for encouraging and motivating employees to improve their performance and achieve positive patient safety outcomes (Arnulf & Larsen 2015). Nurse managers expressed deep concern about the challenges confronting their leadership in terms of patient safety. Inadequate human resources impact on leadership abilities as staff patient ratio compromises patient efficient health service delivery. Kutney-Lee et al. (2016) believe that autonomy in staffing ratio decisions, considering high volumes and acuity levels, will lead to less burnout and a desire to leave the workforce.

Concerning insufficient supplies and equipment, South Africa inherited a centralised procurement model in which the tendering process was managed by the Department of State Expenditure (Moeti 2014). Material resource procurement for hospitals is a component of government service delivery. It is about acquiring goods and services at the lowest total cost of ownership possible, in the right quantity and quality, at the right time, and in the right place, generally through a contract (Ambe & Badenhorst-Weiss 2012). However, centralised procurement and purchasing of supplies and equipment in healthcare institutions disrupts nurse managers’ responsibility for resource management.

### Strengths and limitations

The study revealed nurse managers’ perceptions of the leadership that drives patient safety in an academic hospital, as well as the challenges they face in the process. This was a single qualitative research study conducted in a specific geographical area at an academic hospital. As a result, the detailed description of the study method developed may be replicated in another setting with characteristics like the setting where the study was conducted.

### Recommendations

The study’s findings suggest that staff development activities such as training workshops or refresher courses on leadership skills that promote patient safety be continued. Again, a larger-scale quantitative study may help to highlight leadership-related issues that influence patient safety in hospitals. The findings could lead to the creation of a certificated comprehensive leadership and patient safety competency programme. This type of programme could be part of the mandatory requirements for the consideration for hospital managerial positions at all levels.

## Conclusion

The nurse managers were able to share their critical leadership styles and learning requirements for driving patient safety. Their perceptions and needs are consistent with those found in the literature. The findings will benefit all stakeholders involved in improving patient safety in hospitals by strengthening nurse managers’ leadership styles.
